# Dual slag filters for enhanced phosphorus removal from domestic waste water: performance and mechanisms

**DOI:** 10.1007/s11356-017-0925-y

**Published:** 2017-12-26

**Authors:** Minyu Zuo, Gunno Renman, Jon Petter Gustafsson, Wantana Klysubun

**Affiliations:** 1 Division of Land and Water Resources Engineering, KTH (Royal Institute of Technology), Teknikringen 76, 100 44 Stockholm, Sweden; 2 Department of Soil and Environment, Swedish University of Agricultural Sciences, Box 7014, 750 07 Uppsala, Sweden; 3 Synchrotron Light Research Institute, 111 University Avenue, Muang District,, Nakorn Ratchasima, 30000 Thailand

**Keywords:** Metallurgical slags, Calcium phosphate, Phosphate speciation, Precipitation, Adsorption, Metal release

## Abstract

The phosphorus (P) removal of five combinations of dual filters consisting of blast furnace slag (BFS), argon oxygen decarburisation slag (AOD) and electric arc furnace slag (EAF) was evaluated in column experiments with domestic waste water. The columns were fed with waste water for 24 days. The column with only EAF had the best P removal performance (above 93% throughout the experiment). The speciation of the bound P was evaluated by P K-edge X-ray absorption near-edge structure (XANES) spectroscopy. In all five columns, the main P species of the slag packed in the outlet chamber was amorphous calcium phosphate (ACP). In samples from the inlet chambers, the contributions from crystalline Ca phosphates, P adsorbed on gibbsite and P adsorbed on ferrihydrite were usually much greater, suggesting a shift of P removal mechanism as the waste water travelled from the inlet to the outlet. The results provide strong evidence that P was predominantly removed by the slags through the formation of ACP. However, as the pH decreased with time due to the progressively lower dissolution of alkaline silicate minerals from the slag, the ACP was rendered unstable and hence redissolved, changing the P speciation. It is suggested that this process strongly affected the lifespan of the slag filters. Of the slags examined, EAF slag had the best P removal characteristics and BFS the worst, which probably reflected different dissolution rates of alkaline silicates in the slags.

**Electronic supplementary material:**

The online version of this article (10.1007/s11356-017-0925-y) contains supplementary material, which is available to authorized users.

## Introduction

It is widely known that excess phosphorus entering the water body could lead to eutrophication and thus become a threat to human well-being (Smith 2003). The use of various types of slag to remove excess phosphorus from waters has been studied worldwide in recent years (Kostura et al. 2005; Drizo et al. 2006; Barca et al. 2012; Zuo et al. 2015). Two of the most widely investigated slags for this purpose are the blast furnace slag (BFS) and the electric arc furnace slag (EAF). Both types of slag have been tested in the laboratory as well as on a field scale in different countries and have shown a promising P removal performance. BFS is used as a filter material in constructed wetland systems to remove P and has achieved good P removal performance (Sakadevan and Bavor 1998). It has been suggested that the use of EAF in constructed wetlands is a promising solution for P removal (Drizo et al. 2006). In addition to these two types of slag, argon oxygen decarburisation slag (AOD) has proved to be a potential waste water purification material in our previous research (Zuo et al. 2015).

Precipitation of calcium phosphates (Ca-P) has been reported as the main P removal mechanism of these types of slag owing to their high calcium content and alkalinity (Barca et al. 2012). Many slags have an alkaline reaction in water due to the dissolution of gehlenite (Ca_2_Al_2_SiO_7_) and other silicate minerals (Kostura et al. 2005; Gustafsson et al. 2008), which create favourable conditions for Ca-P precipitation. Possible Ca-P precipitates include hydroxyapatite (Ca_5_(PO_4_)_3_OH; HAP), octacalcium phosphate (Ca_8_H_2_(PO_4_)_6_·5H_2_O; OCP), dicalcium phosphate dehydrate (CaHPO_4_·2H_2_O; DCPD) and amorphous calcium phosphate (Ca_3_(PO_4_)_2_; ACP). Claveau-Mallet et al. (2012) used EAF as a filter to remove P from synthetic waste water and found that the main Ca-P phase accumulated on the slag surface, after 2 years of operation, was apatite. Consistent with this, Barca et al. (2012) propose HAP to be the main product of P precipitation on the surface of EAF and basic oxygen steel slag (BOF). However, Valsami-Jones (2001) states that an amorphous Ca-P phase was formed initially and then transformed into crystalline HAP with time. The results obtained by energy-dispersive X-ray spectroscopy (EDX) and X-ray powder diffraction (XRD) on samples from a 2-year column experiment suggested that the P mineral precipitates on the surface of BOF slag changed from brushite to OCP and then to a mixture of OCP and HAP (Bowden et al. 2009). The discrepancy of the reported P speciation results highlights a need to further investigate the mechanisms governing the formation of different P phases in the slag material.

Further, it has been reported that extensive pretreatment is important to achieve optimum P removal and lifespan of the P removal materials (Nilsson et al. 2013). Therefore, in the present study, the performances of five binary combinations of three different types of slag packed in columns with two identical chambers (‘dual filters’) were compared to determine the set-up leading to the longest lifespan. Dual filters, which consist of an inlet chamber (‘pretreatment filter’) and an outlet chamber (‘polishing filter’) in series, are considered to enhance P removal and prolong the lifespan of the slag material. The slag packed in the inlet chamber was intended to have the following functions: (1) to partly remove the organic matter that would interfere with the P removal process, (2) to increase the pH of the waste water to a point that is favourable for the Ca-P precipitation and (3) to provide extra Ca for the Ca-P precipitation. The material in the second chamber was then expected to retain the Ca and extend the P removal through precipitation.

The objective of the current study was not only to compare P removal performances, but also to investigate the P removal mechanisms of these types of slag, which could give further insight into the factors affecting the lifespan. Hence, P speciation was studied by X-ray absorption near-edge structure (XANES) spectroscopic analysis of the used slag material and linear combination fitting (LCF) of the XANES spectra.

Since concentrations of heavy metals, such as Cr, are elevated in the slag materials compared with those in most soils, there has been growing concern in recent years regarding heavy metal release from the slag material to the environment (Proctor et al. 2000; Chaurand et al. 2007; Windt et al. 2011; Baciocchi et al. 2015). Therefore, the release of potentially toxic metals such as Cr, Zn and Pb from the slags during their contact with waste water was investigated.

## Materials and methods

### Materials

Three slags produced in Sweden with particle sizes ranging from 1 to 2.36 mm were used: BFS from SSAB Merox AB in Oxelösund, AOD slag from Outokumpu Stainless AB in Avesta and EAF slag from Höganäs Sweden AB in Höganäs. All slags were activated by heating at 1000 °C for 15 min before packing to remove the effect of ageing on the P removal performance, as they had been stored in the laboratory for more than 10 months (Zuo et al. 2016a). The chemical composition of the AOD and EAF slags is given in Table 1. The chemical composition of BFS was determined in an earlier study (Johansson Westholm 2010).

**Table 1:** Chemical composition of the used slags (mg g^−1^)

	Si	Mn	P	Cr	Ni	Al	Ca	Fe	Mg
AOD	149.8	3.9	0.04	10.3	0.47	9	380	3	33
BFS	158.6	4.6	nv	nv	nv	68.8	214.3	3.7	100.8
EAF	162.4	11.6	0.04	33	0.39	21.7	325	3	50.1

*nv* no value available

Waste water for the column experiment was collected from septic tank effluent serving five families. No other treatment of the waste water was conducted before it was fed to the columns. The waste water was stored in a plastic container at room temperature. Six batches of waste water were used during the experiment starting on days 1, 4, 9, 12, 16 and 19, respectively. Sampling and analysis of each batch of waste water before and after feeding were performed as the water quality varied from one batch to another (Table 1S).

### Column experiment

The experiments were conducted in a laboratory at a temperature of 21 ± 2 °C. Five vertical, transparent, plastic columns (20 cm long and 5 cm in diameter) were used. Each column was divided into two identical chambers by a vertical plate that had five small holes in the bottom. The inlet and outlet were located in the centre of the top caps of the first and second chambers, respectively.

The slag was combined in five different ways: (i) BB and BFS, followed by BFS; (ii) BA and BFS, followed by AOD; (iii) BE and BFS, followed by EAF; (iv) AA and AOD, followed by AOD; and (v) EE and EAF, with EAF. The two components of each combination were separately packed in two identical chambers of a column. Every chamber was packed with 150 mL slag in the same way (Table 2). The weights were 195.5, 124.5 and 253.5 g for AOD, BFS and EAF, respectively. The bottom and top of the columns were covered with rubber caps and then sealed with silicone to prevent leakage of water and air.

**Table 2:** Composition and loading of columns

Column	Chamber 1	Chamber 2	Inflow rate (mL min^−1^)	Received water (L)	Pore volumes
BB	BFS	BFS	4.15	29.1	117
BA	BFS	AOD	4.15	22	95
BE	BFS	EAF	4.15	28	135
AA	AOD	AOD	4.15	28	135
EE	EAF	EAF	4.15	21.7	116

*BB* column filled with only BFS, *BA* filled with BFS in the first chamber and AOD in the second chamber, *BE* filled with BFS in the first chamber and EAF in the second chamber, *AA* filled with only AOD, *EE* filled with only EAF

Waste water was pumped into the first chambers of the six columns by two peristaltic pumps with six channels; each channel had a feeding rate of 4.15 mL min^−1^. Waste water flowed down the first chamber, passed through the holes at the bottom of the separating plate, then flowed upwards through the second chamber and reached the outlet. The outlet water was collected in five Pyrex media bottles. Effluents from each column were sampled once a day to measure pH, P, dissolved organic carbon (DOC), inorganic carbon (IC) and Ca, Cr, Zn and Pb concentrations. Therefore, these samples were a mixture of several pore volumes of effluent in a day. After sampling, the bottles were emptied and cleaned before being connected to the columns again.

A preliminary experiment was run to determine the duration of pumping. The waste water travelling times from the inlet to the outlet were 45, 60, 55, 50 and 50 min for columns EE, BB, BA, BE and AA, respectively. A pumping duration of 45 min was chosen for every feed so that the waste water in all five columns could be in contact with the slag material until the next feed. The columns were fed in a sequential fashion by a 45-min feed followed by a break for 2 to 3 h (cf. below) during which the pumps were stopped, and then the next feed started. The P removal properties of the slags had previously been determined in a preliminary set of batch experiments during which real waste water was used (data not shown). These results showed that more than 99% of P in the waste water was removed in 4 h. Therefore, the columns were fed six times a day for the first 8 days and then eight times a day until the end of the experiment to accelerate the exhaustion of the slag. The experiment lasted for 24 days, including two breaks (days 3–4, day 7) due to a shortage of waste water. Column BA was not in operation between days 11 and 15 because of a leak at the bottom of the column. Column EE was ended 4 days earlier than the other columns, also because of leakage.

After the experiment, the slag was removed from the columns and mixed thoroughly, chamber by chamber, before sampling. The slag samples were then air-dried for 48 h in the fume hood and ground to fine powder with a mortar prior to XANES analysis.

### Analysis method

Determination of P as molybdate-reactive phosphate was conducted using a Seal Analytical AA3 autoanalyser. The pH was determined with a Hach pH meter (Sension™ pH 31). Inductively coupled plasma optical emission spectrometry (ICP-OES, Thermo Scientific Icap 6000) analysis was conducted to determine the concentrations of Ca, Zn, Cr and Pb. The detection limit of this instrument was ≤ 1 ppb. DOC and IC were analysed using a TOC-L analyser (Shimadzu, Japan).

The P K-edge XANES spectra of the ten ground solid samples were collected in the same way as reported in previous research on beamline BL-8 of the Synchrotron Light Research Institute, Thailand (Klysubun et al. 2012; Zuo et al. 2015). The beamline was equipped with an InSb(111) double crystal monochromator, giving a beam flux of 1.3 × 10^9^ to 6 × 10^10^ photons s^−1^ (100 mA)^−1^ in a 17.7 × 0.9 mm^2^ beam (Eriksson et al. 2016a). Depending on the level of noise in the data, between two and six scans per sample were collected.

The XANES spectra were processed with Athena (version 0.9.24) in the Demeter software package (Ravel and Newville 2005). Poor scans were discarded. The energy was calibrated by setting the maximum of the first derivative of the spectrum for elemental P powder (*E*_0_) to 2145.5 eV, and the correction of energy shift was conducted as in previous research (Zuo et al. 2015). Then, multiple spectra were merged and the merged scans were normalized (Zuo et al. 2015). A normalisation range between 30 and 45 eV was used. However, for the sample from the first chamber of column BB, a normalisation range between 32 and 60 eV was used.

A set of spectra of known standards, measured at BL-8 in the same experimental conditions (Eriksson et al. 2016a, b), was combined to fit the sample spectra using a linear combination fitting approach (Tannazi and Bunker 2005). The fitting range was set from − 10 to 30 eV relative to *E*_0_. Three standards were included in each fit. Fits with weighting fractions summed to 100 ± 10% were accepted. Apart from the 31 standards collected in the database, a new standard called ‘ACP-slag’ was included for the LCF analysis. This standard was the sample AOD-P from the previous paper, in which it was suggested that the AOD-P sample is dominated by an amorphous calcium phosphate phase not covered by the 31 mineral standards that were used (Eriksson et al. 2016a, b).

## Results and discussion

### P removal performance of the slags and pH change

**Fig. 1:**
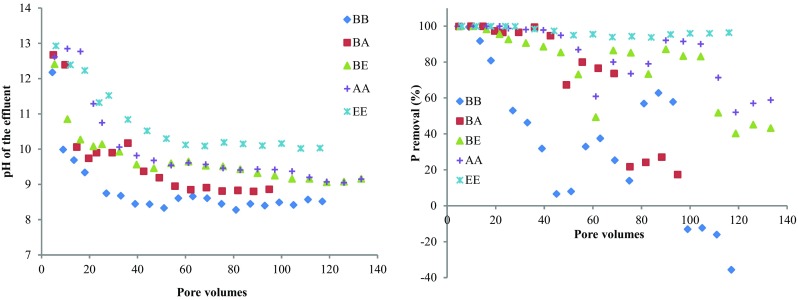
pH of the effluents and the P removal percentage of the columns

Column EE with only EAF slag had the highest P removal among all the columns (Fig. 1). It captured almost 240 mg P out of the 248.9 mg added as its P removal was 100% for the first 28 pore volumes and then fluctuated between 93 and 98% in the following pore volumes. Column AA had a P removal higher than 94.9% for the first 28 pore volumes, and then, the P removal fluctuated between 60.9 and 90% until the end of the experiment. This was unexpected as the batch experiment with synthetic P solution had shown the AOD slag to have a better P removal performance than EAF when they were dosed with the same amount (Zuo et al. 2016b). A possible explanation is the higher bulk density of the EAF slag, as 507 g EAF was packed in column EE, whereas 391 g AOD was packed in column AA. The best P removal performance of column EE was accompanied by the highest effluent pH observed among the five columns. Another possible reason might be the higher pH in column EE, which suggests that the dissolution of alkaline silicates was greater in EAF slag, giving rise to more favourable conditions for Ca-phosphate precipitation.

Column BA had strong P removal for the first 44 pore volumes. A leakage then occurred at the bottom of the column, and it was stopped for 4 days. After this break, the P removal started to decrease. During the first 44 pore volumes, the P removal performance of column BA was higher than that of column BE, but after the leakage, column BA showed less satisfying P removal than column BE. This inferior P removal might be attributed to the enhanced ageing effect caused by carbonation during the 4-day break. The waste water in column BA was drained out after leakage was noticed, then the column was put upside down for 4 days. Through the bottom of column BA where leakage occurred, air could enter and be brought into contact with the wet materials in the column. The CO_2_ of the air could react with the wet calcium-rich slag material through carbonation and accelerate the ageing as reported by Akbarnejad et al. (2012, 2014). During the carbonation process, the carbonation product CaCO_3_ formed a coating. The coating would prevent Ca diffusion from the steel slag to the solution when the column was fed with waste water again (Huijigen et al. 2005, Huijgen and Comans 2006), which in turn would decrease the extent of Ca-P precipitation. Meanwhile, the carbonation process reduced the basicity of the AOD slag (Salman et al. 2014), which might also have contributed to the inferior P removal of column BA after the leakage.

Column BB had the poorest P removal performance, with a rapid decrease in P removal efficiency from 100 to 6.6% during the first 45 pore volumes. It was the only column in which breakthrough was observed. The effluent pH of column BB was the lowest of all columns, decreasing to around 8.5 after pore volume 33, accompanied by a decrease in P removal from 31.9 to 6.6%. Sindelar et al. (2015) report that at a pH of 8.5, Ca precipitation ceased after the addition of 0.5 mg DOC L^−1^. Since the DOC of the waste water was much higher than 0.5 mg L^−1^, it is quite possible that the Ca-P precipitation was inhibited by the presence of DOC.

Similar P removal performance of EAF was reported in other studies using synthetic P solution and different hydraulic retention times (HRTs) (24 h for Drizo et al. 2006, 3.8 h for Claveau-Mallet et al. 2012). But, comparing with column EE in this experiment, the reduction of P removal was much faster in the field experiment conducted by Barca et al. (2013), though a longer HRT was used (24 h). One reason of the faster P removal reduction was because the EAF slag was filled in filter beds open to atmosphere (Barca et al. 2013), which facilitated the carbonation of the slag materials. Besides, Liira et al. (2009) reported that the overall P removal performance of filter materials would decrease with increasing retention time due to chemical clogging caused by carbonate precipitates. Therefore, in order to achieve high P removal and optimal overall P removal performance, the HRT is suggested to be between 4 and 6 h for using slags to remove P from waters.

### Effluent and influent Ca concentrations

**Fig. 2:**
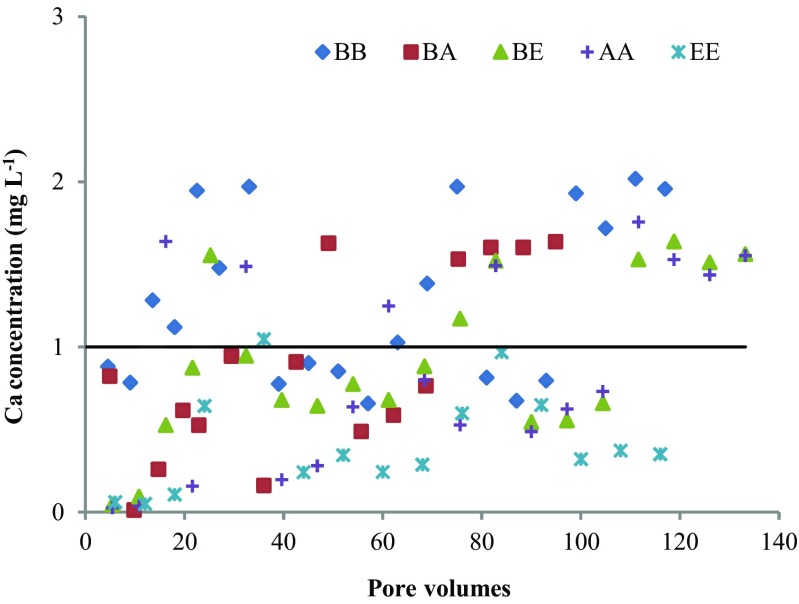
The ratio of the effluent to the influent Ca concentrations as a function of pore volumes

The Ca concentration of the influent varied between 20.0 and 42.5 mg L^−1^ depending on the waste water batch. The effluent and influent Ca concentration ratio as a function of pore volumes is shown in Fig. 2. The effluent from column EE had the lowest Ca concentration among the five columns, while the effluent from column BB had the highest Ca concentration, which was also higher than that of the influent most of the time. The other four columns had effluents with lower Ca concentrations than those of the influent most of the time, which means that the influent Ca probably acted as a Ca source for precipitation of Ca-P phases in these four columns.

The temporal development of the effluent Ca concentrations showed an opposite trend compared to the pH change. The Ca concentrations of the effluent were very low, even negligible, during the first 20 pore volumes for columns BE, AA and EE. They then increased gradually with time and eventually became higher than those of the influent. Engström et al. (2014) report that a large amount of Ca was released from three EAF slag samples at the beginning of the leaching experiment, and the Ca release decreased with time as the slag aged. The opposite trend of effluent Ca concentrations observed in this research may be due to other reactions involving Ca. A very obvious decrease in inorganic carbon was observed for effluents from columns EE, AA and BE at the beginning of the experiment (Fig. 2S), which may be caused by calcite formation, a competitive reaction to Ca-P precipitation, as reported by Claveau-Mallet et al. (2012).

In column AA, large fluctuations in the dissolved Ca concentration were observed at pore volumes 16 and 32, which were partly because of feeding failure so that the waste water stayed in the column for a longer time than intended. Another reason for the sudden increase in Ca concentrations in the effluents at pore volume 32 was that the influent itself had a higher Ca concentration (42.5 mg L^−1^), as shown in Table 1S. Hence, all effluents showed significantly higher Ca concentrations immediately after pore volume 32.

A layer of white flocs was observed on the surface of the second chamber of column BE (Image 1S) after 12 pore volumes. For both columns BA and AA, the white precipitates were evenly distributed on the AOD slag surface throughout the second chamber. Besides, it is also worth noting that the standing waste water in column EE (on the left of Image 2S) was much clearer than that in the other four columns (Images 1S, 2S), suggesting that other pollutants were removed at the same time from the waste water during Ca-P precipitation. This was evidenced by the decrease of the effluent DOC from 44 to around 30 mg L^−1^ for all five columns, possibly due to the coagulation with Ca and other metal ions released from the slag (Aryal et al. 2011).

### Phosphorus speciation and removal mechanisms

**Fig. 3:**
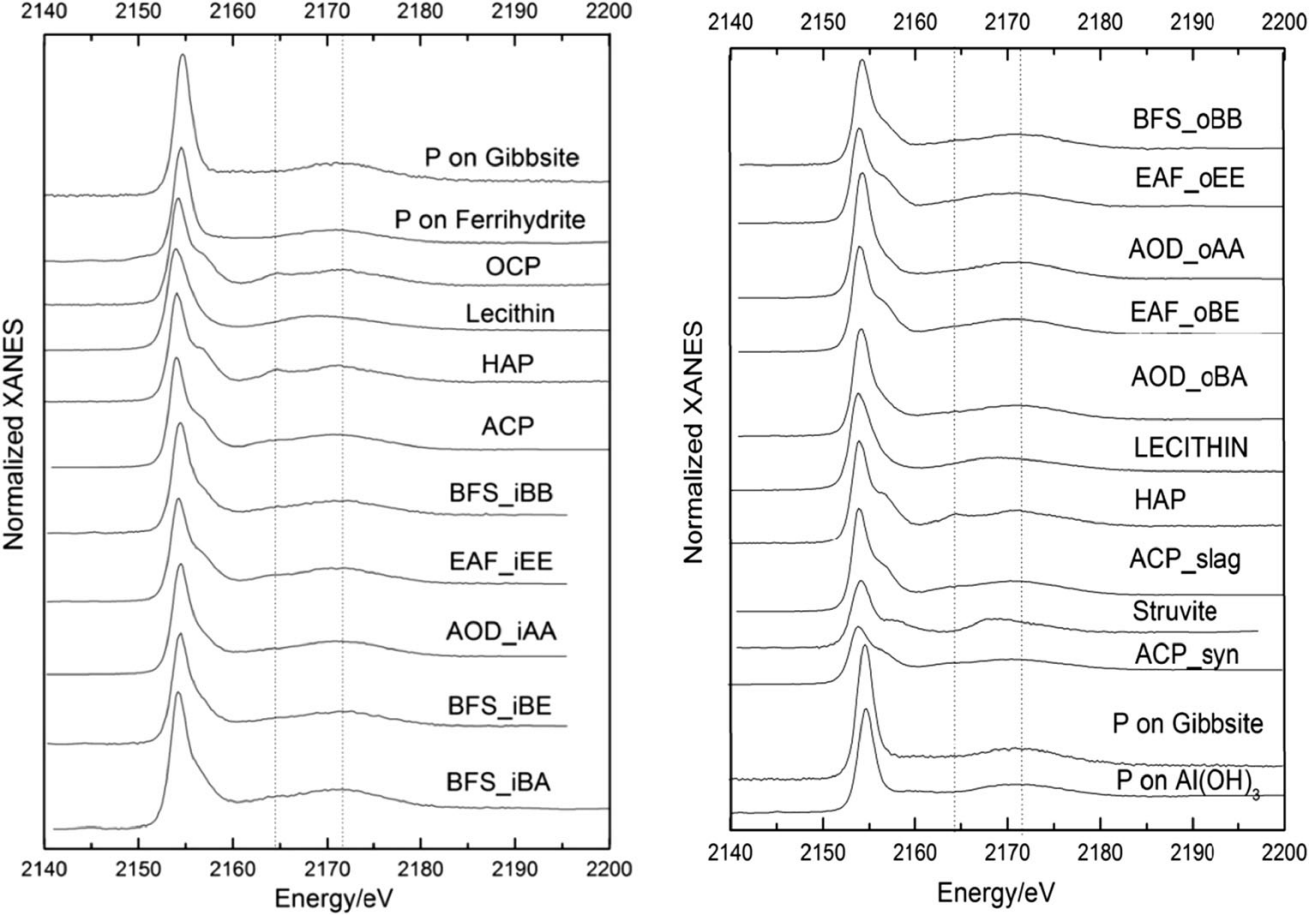
Normalized stacked P K-edge XANES spectra for samples and for the standards of importance in LCF fitting. The dashed lines show the post-white line peaks of apatite at 2164.7 and 2173.3 eV

Stacked P K-edge XANES spectra of all the slag samples and of relevant standards are shown in Fig. 3. Linear combination fitting results of all samples are shown in Table 3 and Fig. 1S. Phosphorus species with estimated amounts below 5% were excluded from the LCF result table because the result may not be reliable (Werner and Prietzel 2015).

**Table 3:** Phosphorus speciation as evidenced from linear combination fitting of XANES spectra

		ACP slag	Gibbsite	P_Al(OH)_3_	Lecithin	HAP	Struvite	ACP_syn	*R* factor
AOD_oBA	Weight (%)	69 ± 3%	23 ± 1%		14 ± 2%				0.003
	Presence	1, 2, 3, 4, 5	1, 3	2, 3, 4, 5	1, 2				
EAF_oBE	Weight (%)	81 ± 1%						13 ± 1%	0.0008
	Presence	1, 2, 3, 4, 5				2, 4	2, 3	1, 3	
AOD_oAA	Weight (%)	62 ± 1%	16 ± 3%	25 ± 4%					0.0020
	Presence	1, 2, 3, 4, 5	1	1, 2, 3, 4, 5					
EAF_oEE	Weight (%)	54 ± 5%				40 ± 7%	7 ± 3%		0.0037
	Presence	1, 2, 3, 4, 5			2, 4	1, 2, 3, 5	1		
BFS_oBB	Weight(%)	81 ± 1%	12% ± 1%		8 ± 1%				0.001
	Presence	1, 2, 3, 4, 5	1, 2, 3, 4, 5		1				
		ACP slag	Gibbsite	OCP	Lecithin	HAP	Ferrihydrite	*R* factor	
BFS_iBA	Weight (%)	80 ± 2%	19 ± 1%		8 ± 2%			0.0016	
	Presence	1, 2, 3, 4, 5	1, 2, 3, 4, 5		1				
BFS_iBE	Weight (%)	10 ± 4%	34 ± 1%	58 ± 5%				0.0026	
	Presence	1	1, 2, 3, 4, 5	1, 2, 3, 4, 5					
AOD_iAA	Weight (%)		20 ± 3%	52 ± 1%			32 ± 4%	0.0024	
	Presence	2, 3, 5	1, 4	1, 2		2, 4	1, 2, 3, 4, 5		
EAF_iEE	Weight (%)	17 ± 4%		70 ± 5%			18 ± 1%	0.0028	
	Presence	1, 4	2, 3, 4, 5	1, 2, 3, 4, 5			1, 2		
BFS_iBB	Weight (%)	11 ± 4%	33 ± 1%	57 ± 5%				0.0022	
	Presence	1	1, 2, 3, 4, 5	1, 2, 3, 4, 5					

*AOD_oBA* AOD slag from the outlet chamber of column BA, *BFS_iBA* BFS slag from the inlet chamber of column BA

There was a difference in P speciation between the samples collected from the inlet chambers and those from the outlet chambers. For all five samples from outlet chambers, the main P species was ACP, while the main P species for BFS_iBA, AOD_iAA and the other three samples collected from the inlet chambers were ACP, P adsorbed on ferrihydrite and OCP, respectively.

It is reasonable to assume that the pH of the waste water increased as the waste water travelled from the inlet chamber to the outlet chamber because the dissolution of the alkaline silicate minerals in the slag would increase the pH. As the pH increases from 7 to 8 or even higher, the main P removal mechanism shifts from adsorption on Fe and Al (hydr)oxides to precipitation as calcium phosphates (Eriksson et al. 2016a, b). The LCF results agreed well with this, as the pH remained above 8 in all columns and as Ca phosphates accounted for the majority of the retained P. For the five samples collected from the inlet chambers, the weight of P adsorbed to gibbsite and ferrihydrite varied from 18 to 52%. However, a weaker contribution (12–41%) of P adsorbed to gibbsite or Al(OH)_3_ was suggested for samples collected in the outlet chambers.

EAF samples collected from the inlet and outlet chambers showed significant differences in the composition of P species (Table 3). The main P species of EAF_iEE was OCP (70%), whereas for both outlet columns EAF_oBE and EAF_oEE, the main P species was ACP (81 and 54%, respectively). However, the post-edge features of samples EAF_oEE and EAF_iEE were not well described by the LCF since the first post-white line peaks of both samples were much weaker than the fit (Fig. 1S). HAP and OCP were included in the LCF results of the two samples, both of which displayed a very obvious post-edge shoulder that was different from the two EAF samples. Therefore, it is probable that these samples contained some Ca-P species not included in the standard list, a species that is more amorphous than HAP and OCP, but more crystalline than ACP.

The P speciation of the three AOD samples was dominated by Ca phosphates, but again, the composition of these was different in the inlet and outlet columns. In the outlet columns, ACP was the predominating P phase with 62 and 69% for AOD_oAA and AOD_oBA, respectively. In AOD_iAA, ACP was not identified; instead, OCP accounted for 52% of the retained P. There was also a relatively strong contribution for P bound to Al hydroxides (41, 23 and 20% for AOD_oAA, AOD_oBA and AOD_iAA, respectively), and the results for AOD_iAA also indicated a role of P bound to ferrihydrite (32%). Lastly, the presence of organic P was indicated for the AOD_oBA sample, due to the inclusion of lecithin (14%) in the LCF result. Lecithin is a diester P compound that has been found to describe organic P well in soils (Eriksson et al. 2016a, b).

As concerns the BFS, the outlet column sample BFS_oBB was dominated by ACP (81%), with minor contributions of P adsorbed on gibbsite and lecithin. The samples from the two inlet columns BFS_iBB and BFS_iBE were very similar and contained mostly OCP (57 and 58%, respectively) and P adsorbed to gibbsite (33 and 34%, respectively), with a small additional contribution of ACP (11 and 10%, respectively). It is very probable that the inlet chamber had much lower pH than the outlet chamber, causing the ACP formed on BFS_iBE and BFS_iBB to dissolve with the decrease in pH at the end of the experiment, leaving mostly OCP and Al-bound P on the slag samples. A similar P speciation was expected also for the third inlet column sample BFS_iBA; however, instead, the P speciation of this sample was similar to that of BFS_oBB, with a predominance of ACP (Table 3). The reason for this divergent result could not be established.

With the exception of the result of the BFS_iBA sample, the results are consistent with an interpretation in which the P removal of all three slag materials occurs primarily through ACP formation, which occurs when the pH is sufficiently high. In the outlet columns, these conditions were met. Part of the ACP may slowly recrystallize to form less soluble Ca phosphates (e.g. OCP, apatite). However, with time, the pH will decrease towards the pH of the infiltrating waste water, which will cause the ACP to dissolve. Again, some of the dissolved P may be reprecipitated as less soluble Ca phosphates (e.g. OCP, apatite), and the lower pH also facilitates a certain amount of P adsorption onto Fe and Al hydrous oxides on the slag surfaces. However, a substantial fraction of the dissolved P will not be resorbed but will instead migrate to the outlet column, where it is reprecipitated again as ACP if the pH is sufficiently high. This suggests a significant role of the stability of ACP for determining both the P removal performance and lifespan of the filter.

This interpretation is in agreement with the hypothesis stated by Eveborn et al. (2009), which was based on observations for six used filter media, for which the P composition varied as a function of pH. As an example, they observed that the P species of a BFS sample that had been fed with a synthetic P solution was mainly Al bound (62%) and OCP (39%) after a pH of 8 was reached, while ACP was an important P phase in many samples for which a higher pH had been maintained.

In some earlier studies (Claveau-Mallet et al. 2012), apatite was observed as the predominant reaction product of P removal by slag. In part, this may be due to the different techniques used for P phase characterisation; for example, X-ray diffraction can only distinguish crystalline Ca-P phases and cannot be used to identify ACP. Another reason may be related to the fact that in this study, real waste water from a septic tank was used. Van der Houwen et al. (2003) found that the crystallinity of the Ca-P precipitate decreases in the presence of organic ligands. Similar results were obtained by Capdevielle et al. (2016), who studied the impact of organic matter on struvite crystallisation. A third possible reason for the formation of less crystalline P species could be the shorter hydraulic retention time. A minimum hydraulic retention time is necessary for raising the pH of the waste water by dissolution of alkaline material in the slag.

### Dissolved Cr, Pb and Zn in the effluent

**Fig. 4:**
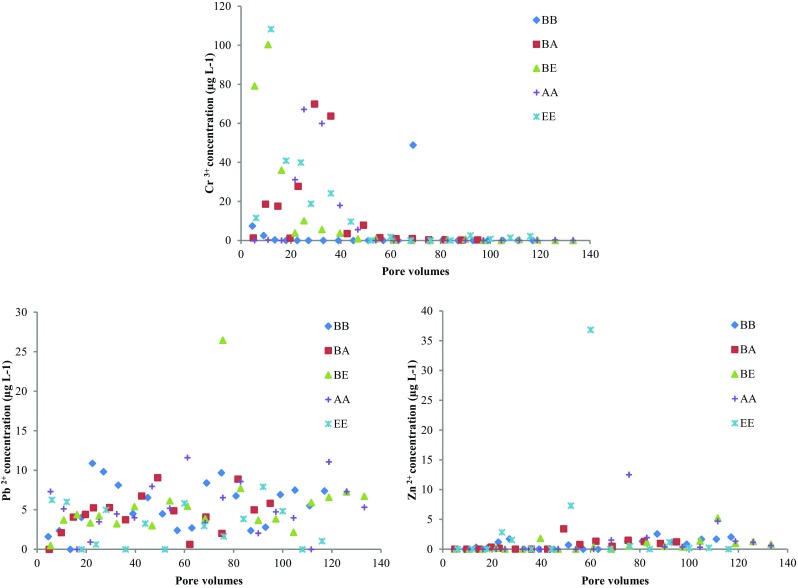
Cr, Pb and Zn concentrations of the effluent as a function of pore volume (liquid to solid ratios of 2 L kg^−1^, according to EN 12457-1:^2002^)

The leaching of Cr, Pb and Zn as a function of pore volumes was calculated at a liquid-to-solid ratio of 2 L kg^−1^ according to EN 12457-1:^2002^. The leaching limit values for Cr, Pb and Zn were 400, 400 and 4000 μg L^−1^, respectively, according to EN 12457-1:^2002^. However, the concentrations shown in Fig. 4 are all significantly below the prescribed limits, indicating no hazardous risk of Cr, Pb and Zn leaching resulting from the use of the slags for water treatment.

The decrease of Cr leaching as a function of the number of pore volumes was observed for columns packed with either EAF or AOD. The Cr leaching of all four columns became negligible from pore volume 40 and onwards, when the pH of all columns dropped below 10.5. This is consistent with the work of Santos et al. (2013), who found that a reduction in Cr leaching from AOD slag could be observed when pH dropped below 10.5. However, other parameters such as the presence of C-S-H, precipitation of double salts and carbonation were also suggested to affect the Cr leaching pattern (Fernandez Bertos et al. 2004; Salman et al. 2014). For columns BA and AA, the release of Cr was less significant than that for the columns BE and EE. Higher Cr concentrations of EAF effluents were also observed by Baciocchi et al. (2015) in the pH range from 8 to 13. The solid-phase Cr contents of EAF and AOD were 33 and 10.3 mg g^−1^, respectively (Table 1), which may partly account for the higher release of Cr from EAF.

## Conclusions

EAF slag had better P removal from domestic waste water in column experiments with a hydraulic retention time of 3 h than the other two types of slag. Being the column with the poorest P removal, column BB was the only one in which breakthrough was observed. The leaching results show no environmental risk of leached Cr, Zn and Pb from the slags.

The P speciation in all outlet columns was dominated by ACP. In the inlet columns, however, ACP was only a minor phase (with the exception of BFS_iBA). These results show that for all dual filters studied, the P was removed primarily as ACP. As a result of successive acidification of the slags due to lower silicate mineral dissolution, ACP was rendered thermodynamically unstable, which caused a changed P speciation and an increased leakage of P from the inlet column to the outlet column. Therefore, based on these results, we hypothesize that the lifespan of the slag filters is intimately linked to the stability of ACP.

## Electronic supplementary material

ESM 1 (DOC 1767 kb)

## Abbreviations

AA: column packed with AOD in both the inlet chamber and the outlet chamber
ACP: amorphous calcium phosphate
AOD: argon oxygen decarburisation slag
AOD_iAA: used AOD from the inlet chamber of column AA
AOD_oAA: used AOD from the outlet chamber of column AA
AOD_oBA: used AOD from the outlet chamber of column BA
BA: column packed with BFS in the inlet chamber and AOD in the outlet chamber
BB: column packed with BFS in both the inlet chamber and the outlet chamber
BE: column packed with BFS in the inlet chamber and EAF in the outlet chamber
BET: Brunauer-Emmett-Teller
BFS: blast furnace slag
BFS_iBA: used BFS from the inlet chamber of column BA
BFS_iBB: used BFS from the inlet chamber of column BB
BFS_iBE: used BFS from the inlet chamber of column BE
BFS_oBB: used BFS from the outlet chamber of column BB
DCPD: dicalcium phosphate dehydrate
DOC: dissolved organic carbon
EAF: electric arc furnace slag
EAF_iEE: used EAF from the inlet chamber of column EE
EAF_oBE: used EAF from the outlet chamber of column BE
EAF_oEE: used EAF from the outlet chamber of column EE
EE: column packed with EAF in both the inlet chamber and the outlet chamber
EDX: energy-dispersive X-ray spectroscopy
HAP: hydroxyapatite
IC: inorganic carbon
ICP-OES: inductively coupled plasma optical emission spectrometry
LCF: linear combination fitting
OCP: octacalcium phosphate
PEG: polyethylene glycol
XANES: X-ray absorption near-edge structure spectroscopy
XRD: X-ray powder diffraction
