# Supplementary material for: Dual slag filters for enhanced phosphorus removal from domestic waste water: performance and mechanisms
Source: Environ Sci Pollut Res Int. 2017 Dec 26;25(8):7391–400. doi: 10.1007/s11356-017-0925-y (PMC5847628; doi:10.1007/s11356-017-0925-y)
Supplement: Supplementary file 1 — (DOC 1767 kb) [file 11356_2017_925_MOESM1_ESM.doc]

**Supplementary tables and figures**

**Dual slag filters for enhanced phosphorus removal from domestic wastewater: performance and mechanisms**

**Minyu Zuo^a^ *, Gunno Renman^a^, Jon Petter Gustafsson^a,b^, Wantana Klysubun^c^**

**^a^Division of Land and Water Resources Engineering, KTH (Royal Institute of Technology), Teknikringen 76, SE-100 44 Stockholm, Sweden (**[minyu@kth.se](mailto:minyu@kth.se) , [gunno@kth.se](mailto:gunno@kth.se) )

**^b^Department of Soil and Environment, Swedish University of Agricultural Sciences, Box 7014, SE-750 07 Uppsala, Sweden (**[jon-petter.gustafsson@slu.se](mailto:jon-petter.gustafsson@slu.se))

**^c^Synchrotron Light Research Institute, 111 University Avenue, Muang District, Nakorn Ratchasima 30000, Thailand (**[wantana.slri@gmail.com](mailto:wantana.slri@gmail.com))

***Corresponding author, +46 08 790 6567**

**Table 1S. C**hemical characteristics of the wastewater

| Collected time (day) | Ca^2+^ (mg L^-1^) | DOC | IC | P-PO_4_^3-^ (mg L^-1^) | pH |
| --- | --- | --- | --- | --- | --- |
| 1st | 39.5 | 44.28 | 74.62 | 18.36 | 7.52 |
| 4th | 25.6 | 48.12 | 86.42 | 10.6 | 7.81 |
| 9th | 42.5 | 13.95 | 75.19 | 7.44 | 7.57 |
| 12th | 23.8 | 27.16 | 81.57 | 8.8 | 8.13 |
| 16th | 37.5 | 28 | 84.6 | 17.07 | 7.72 |
| 19th | 20.04 | 28.85 |  | 8.47 | 8.04 |

DOC: dissolved organic carbon (mg L^-1^)

IC: inorganic carbon (mg L^-1^)


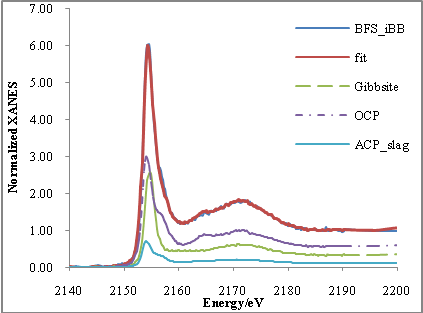

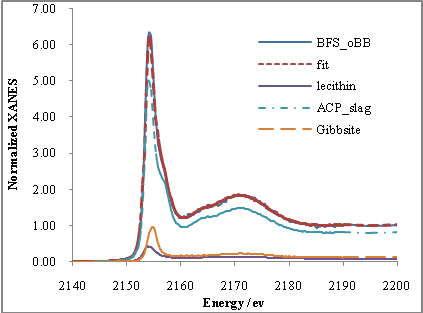


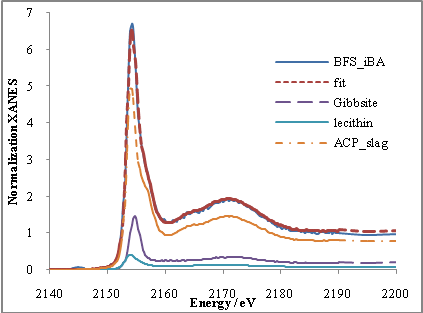

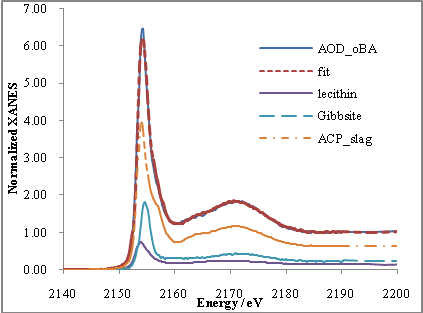


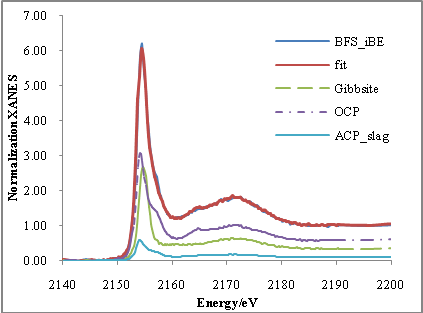

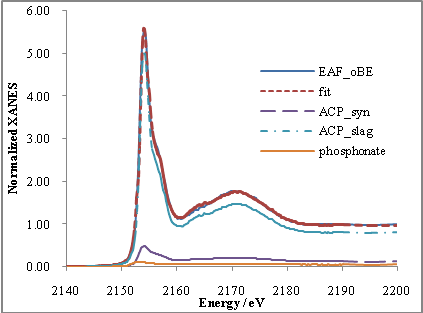


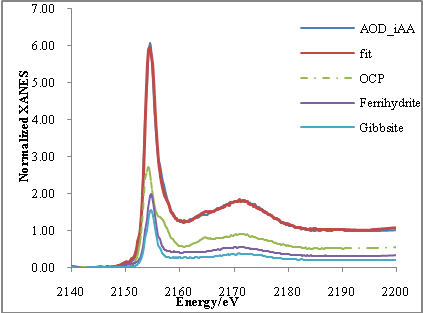

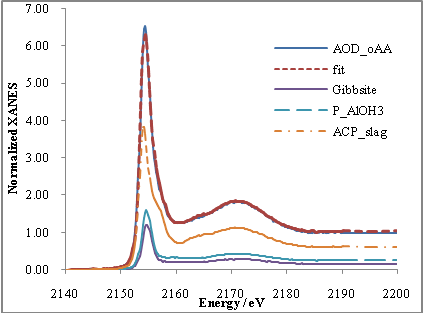


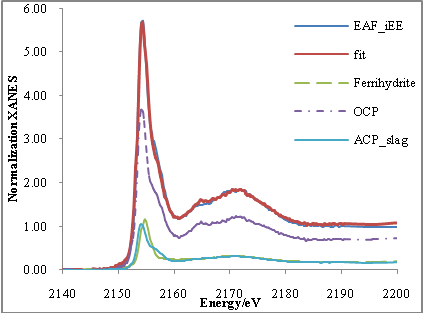

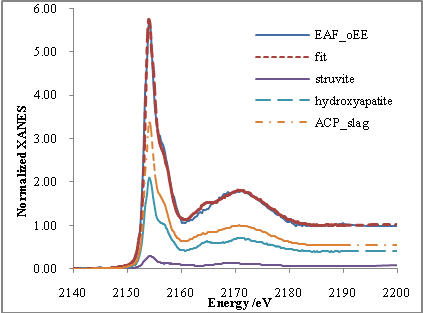


**Figure 1S. Linear combinations and normalized sample data for the ten solid samples**


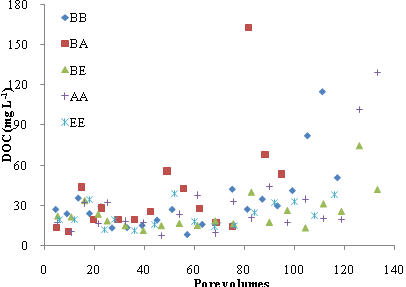

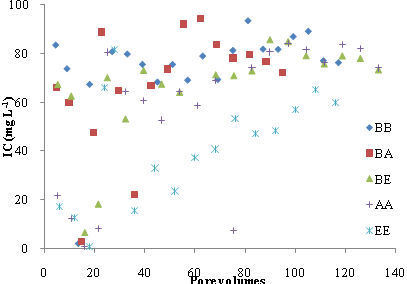


**Figure 2S DOC and IC of the effluents from the five columns**


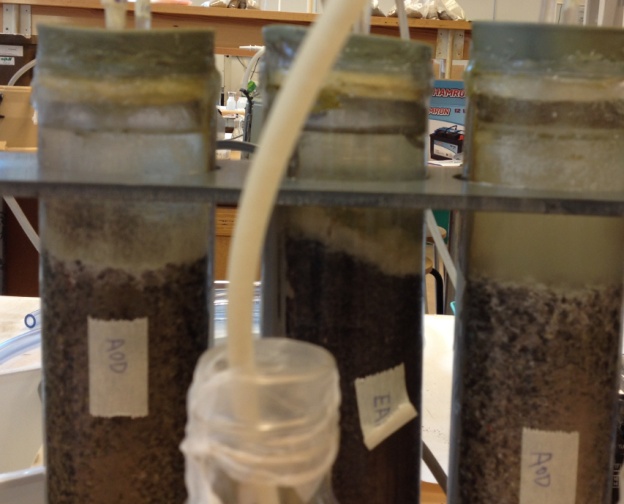


**Image 1s. Flocs in outlet chamber of column BE (middle) and white precipitates in column BA (left) and AA(right).**


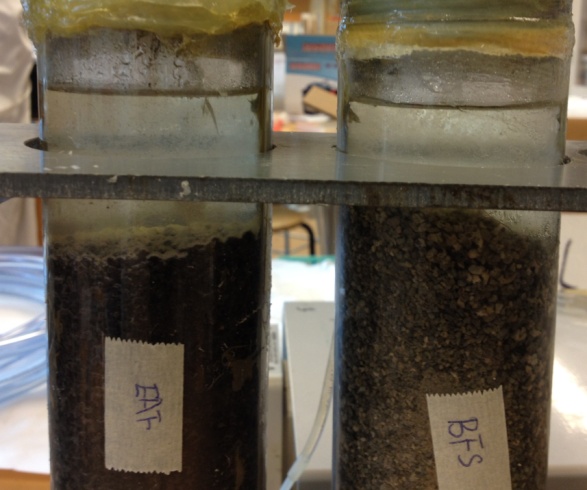


**Image 2s. column EE (left) and column BB(right) after fed with 12 pore volumes.**
